# Supplementary material for: Ultra-sensitive nanometric flat laser prints for binocular stereoscopic image
Source: Nat Commun. 2021 Feb 19;12:1154. doi: 10.1038/s41467-021-21499-4 (PMC7896083; doi:10.1038/s41467-021-21499-4)
Supplement: Supplementary file 1 — Supplementary Information [file 41467_2021_21499_MOESM1_ESM.pdf]

Supplementary information

Ultra-sensitive nanometric flat laser prints for binocular stereoscopic image

Dejiao Hu[1#], Hao Li[1#], Yupeng Zhu[2], Yuqiu Lei[1],Jing Han[1], Shilin Xian[2] , Jiajin Zheng[3], Bai-Ou Guan[1], Yaoyu Cao[1*], Lei Bi[2*] and Xiangping Li[1*]

[1]Guangdong Provincial Key Laboratory of Optical Fiber Sensing and Communications, Institute of Photonics Technology, Jinan University, Guangzhou, China, 510632

[2]National Engineering Research Center of Electromagnetic Radiation Control Materials, University of Electronic Science and Technology of China, Chengdu, China, 610054

[3]College of Electronic and Optical Engineering & College of Microelectronics, Nanjing University of Posts and Telecommunications, Nanjing, China, 210046

[#] These authors contributed equally
*Corresponding author: xiangpingli@jnu.edu.cn; bilei@uestc.edu.cn; yaoyucao@jnu.edu.cn

Supplementary Note 1: Interfacial phase shifts induced ultra-sensitive resonance manipulation

The sensitivity of the Fabry-Perot (FP) resonance wavelength to its thickness is discussed. In such a configuration (schemed in Supplementary Fig.4a), the phase retardations in one round-trip include both propagation phase shifts in the cavity and interfacial phase shifts acquired at two boundaries. The resonance condition can be matched when the resonance wavelength is related to the FP thickness as following

$$\lambda = \frac{2n}{m-(\frac{\varphi_1+\varphi_2}{2\pi})} h \qquad (1)$$

where *n* is the real part of the refractive index of the medium in the FP cavity, *h* is its thickness, m is the resonance order. φ_1 and φ_2 are the interfacial phase shifts defined to lie in the interval [0, 2π) at the two interfaces originating from the imaginary parts of index, respectively. It is shown that the sensitivity of the resonance wavelength to the FP thickness is mainly determined by the refractive index and the total interfacial phase shift at the interfaces. For lossless dielectrics, the interfacial phase shifts are usually restricted to 0 or π, depending on the refractive index contrast at the interface[1]. The propagation phase accumulation in a round trip linearly proportional to the thickness is dominant over the interfacial phase shifts for resonance manipulation. It is revealed that the resonance wavelength has a slow and linear variation along with the FP thickness if the contribution from total interfacial phase shifts is negligible. However, when the total phase shift approaches to 2π, the sensitivity curve of the first order resonance increases exponentially, indicating an ultra-sensitivity, as illustrated in Supplementary Fig.4. In this case, total interfacial phase shifts dominantly

contribute to the first order resonance manipulation and the propagation phase shifts become relatively negligible. Thus, the resonance wavelength showcases a large tunability even the FP thickness is far smaller than the wavelength ($<\lambda/20$).

To this purpose, materials with large both real and imaginary parts of indices as well as proper substrate designs play an important role to realize an ultra-sensitive resonance manipulations[2, 3, 4, 5], holding tantalizing potentials to realize ultra-sensitivity. The total interfacial phase shifts for MoS_2 on different substrates are calculated by using the Fresnel formulas, as shown in Supplementary Fig.5. It can be seen that the total interfacial phase shift of the MoS_2-Au pair manifests a broadband response near 2π in the visible regime, outperforming the other substrates such as Ag and Silicon. By varying the thickness of MoS_2 layers, the ultra-sensitivity of resonance wavelength manipulation supported in the MoS_2-Au configuration reveals the largest tunability in reflectance spectra for wide color palettes as shown in Supplementary Fig.5.

For comparison, the sensitivity of different materials integrated on the Au substrate is calculated in Supplementary Fig.6. Clearly, the MoS_2-Au configuration outperforms the other materials by showing an ultra-sensitivity of 13.95 nm per MoS_2 layer, which corresponds to up to nearly 6 times and 2 times larger than that of 2.3 nm per graphene layer and 5.9 nm per Ge layer, respectively. This result is in good congruence with the illustration in Fig. 1c where the refractive indices of MoS_2 layers overlap with the widest range of total interfacial phase shifts close to 2π.

Supplementary Note 2: Calculate heat distribution during laser exfoliation.

The temperature distribution of 15 layers of MoS_2 film on the Au substrate during laser exfoliation is calculated in Supplementary Fig.7. The substrate is set at room temperature and the laser beam with a Gaussian profile is focused by an objective lens (numerical aperture of 0.75) to a diffraction-limited spot with diameter of 430 nm. Three-dimensional temperature distribution of the layered MoS_2 film can be obtained by the following thermal diffusion equation[6].

$$
\begin{aligned}
c_p \cdot \rho \cdot \frac{du_1}{dt} &= I_1 + k_r \frac{d^2u_1}{dr^2} + \frac{k_r \frac{du_1}{dr}}{r} - \frac{k_z(u_1-u_2)+k_{air}(u_1-RT)}{dz^2} \\
c_p \cdot \rho \cdot \frac{du_i}{dt} &= I_i + k_r \frac{d^2u_i}{dr^{\ 2}} + \frac{k_r \frac{du_i}{dr}}{r} - \frac{k_z(2u_i-u_{i-1}-u_{i+1})}{dz^2} \qquad (2) \\
c_p \cdot \rho \cdot \frac{du_f}{dt} &= I_f + k_r \frac{d^2u_f}{dr^2} + \frac{k_r \frac{du_f}{dr}}{r} - \frac{k_z(2u_f-u_{f-1})+k_{sub}(u_f-RT)}{dz^2}
\end{aligned}
$$

Where C_p is the specific heat, ρ is the mass density and I_i is the absorbed light power density of the ith layer. U_i (r, t) is the temperature distribution in the ith layer at the position *r* and time *T*. The electric field distribution within the MoS_2 layers upon focused laser beam illumination was obtained by using the finite difference time domain (FDTD) method. Then the absorbed optical power flow at each point was extracted with the equation $q_{abs} = \frac{1}{2}\omega\varepsilon''|E|^2$, where ω is the angular frequency and ε'' is the imaginary part of the dielectric function $\varepsilon = \varepsilon' + i\varepsilon''$ of the resonator material and used to calculate the temperature distribution by solving the thermal diffusion equations. In the calculation, the time step is set $dt = 0.1\,ns$ and the spatial step is set $dz = 0.67\,nm$. The specific parameters are shown in Table S1.

Supplementary Fig.7 illustrates the temperature distribution calculated at the incident amplitude of 1.5 mV/m. The two orders of magnitude difference in the in-

plane and out-of-plane thermal conductivity[7, 8] is responsible for a sharp temperature gradient of 74.4 degrees/nm along stacking layers. Even the initial thickness of the layered film of 20 nm is within the focal depth (~ 1.3μm) of the tightly focused beam, it allows precise exfoliation of atop atomic layers without adversely affecting the bottom layers.

Parameter	Value
ρ	$5.1*10^{-12}$(g/μm^3)
R	0.45
k_i	$43*10^{-6}$(W/(μm*k))
k_o	$43*10^{-8}$(W/(μm*k))
k_{air}	$1.204*10^{-8}$(W/(mm*k))
k_{sub}	$320*10^{-6}$(W/(μm*k))
Cp	65.13(J/(mol*k))

Table S1 Parameters used for temperature distribution calculations [7, 9, 10, 11]

	Citation	Thickness/ surface corrugation	Colour gamut	Printing method	Angle tolerance
a	Ref. [12]	30nm-45nm	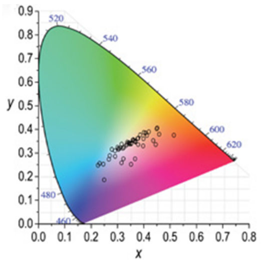	fs Laser	60°
b	Ref.[13]	150nm	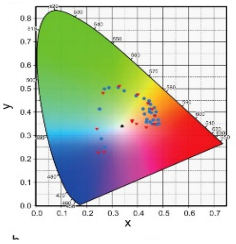	Electron beam lithography	40°
c	Ref.[14]	130nm	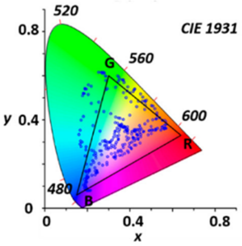	Electron beam lithography	40°
d	Ref.[15]	175nm	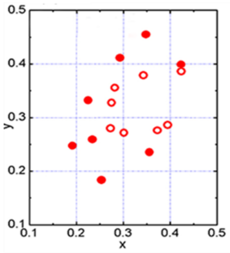	Focused ion beam	70°
e	Our work	15 nm	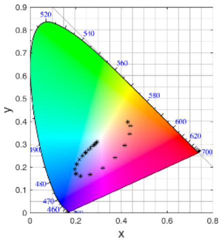	CW laser	60°

Table S2 Comparison of the performance with other structural color papers in terms of structural thickness/surface corrugations, color gamut, printing methods and angle tolerance.

Supplementary Figures

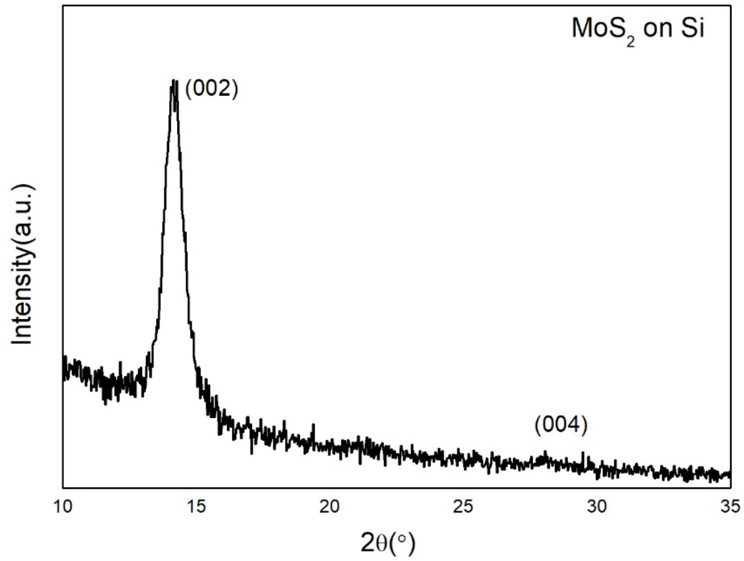

Supplementary Figure 1. X-ray diffraction pattern of an as-grown 10 nm-thick MoS_2 film deposited on SiO2/Si by pulsed laser deposition.

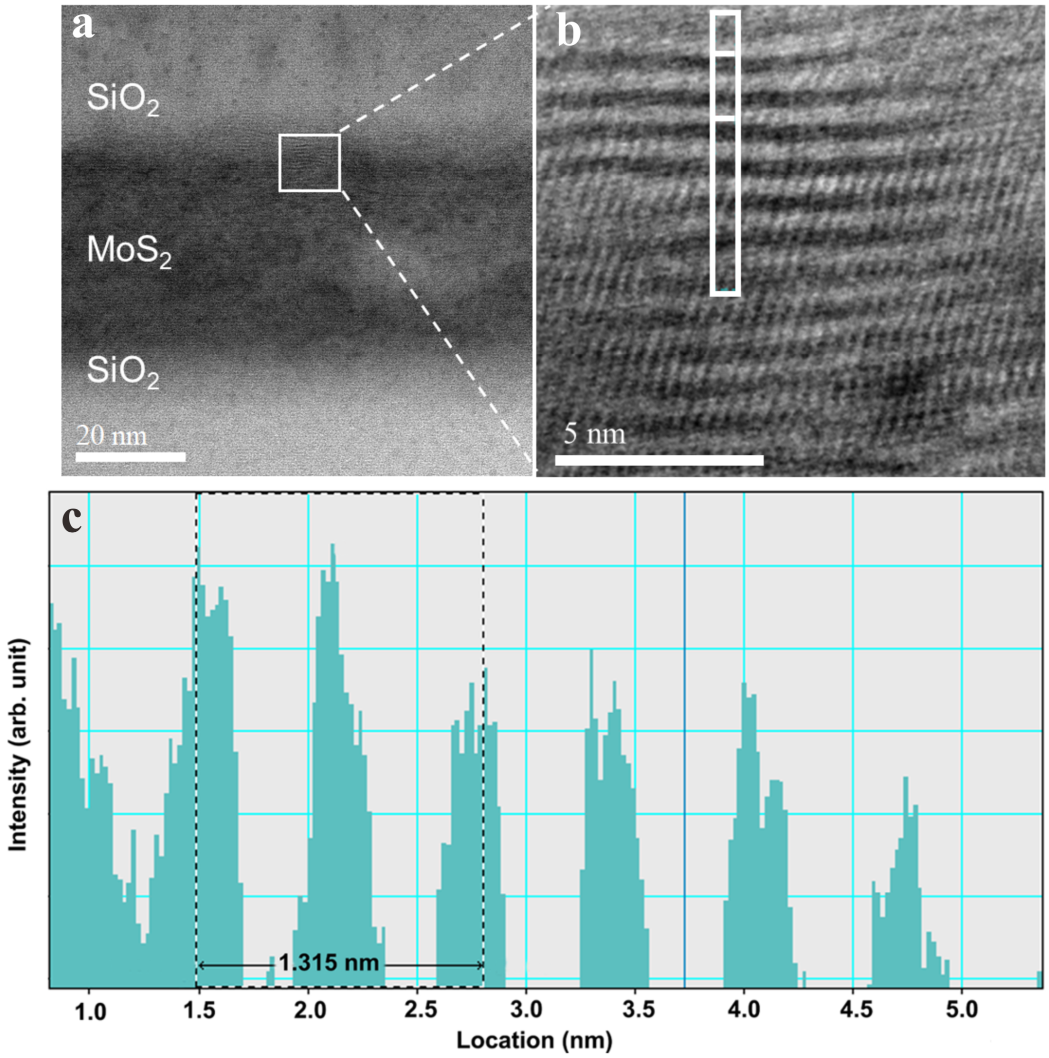

Supplementary Figure 2. (a) STEM image of a MoS_2 thin film grown on SiO_2 (b) HR-STEM imaging showing layered structure of the MoS_2 thin film. (c) Intensity profile of along the cross-section shown in (b)

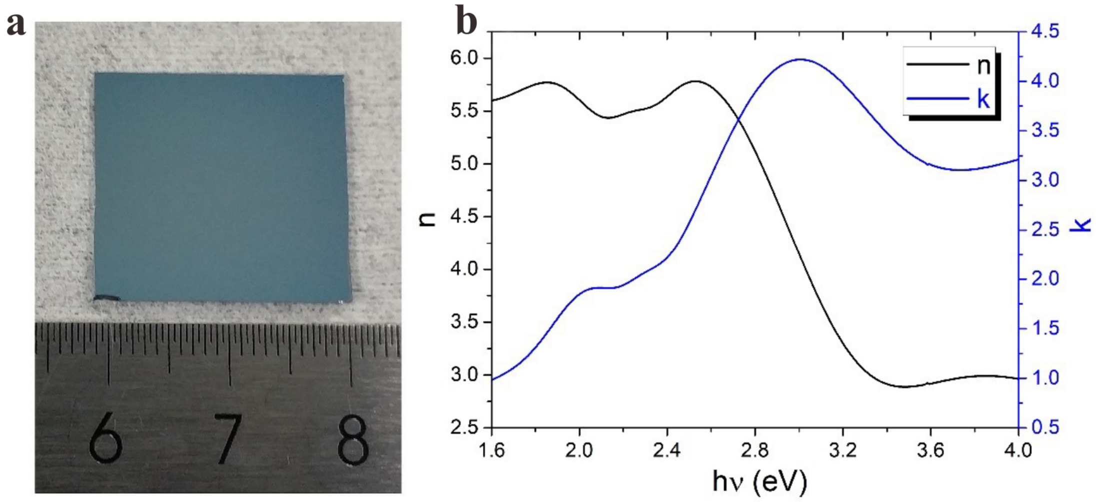

Supplementary Figure 3. (a) Photograph of MoS_2 thin film deposited by PLD on a 2 cm by 2 cm SiO2/Si substrate. (b) Measured optical constants of MoS_2 in the visible to near infrared wavelength range.

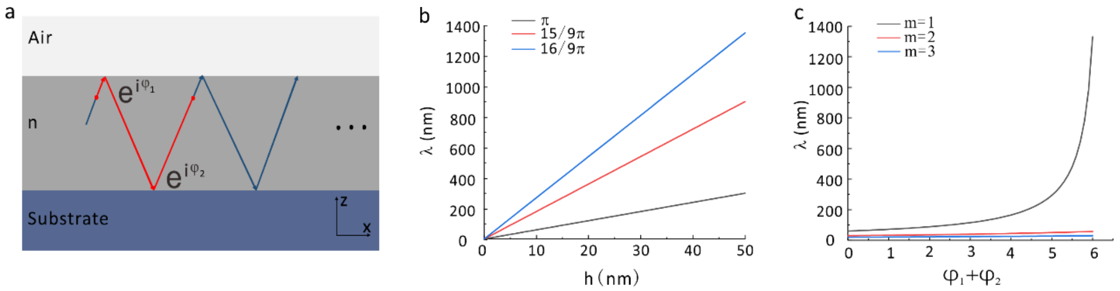

Supplementary Figure 4. The sensitivity of the Fabry-Perot (FP) resonance wavelength to its thickness. (a) Schematic configuration of a FP cavity formed by a dielectric layer with refractive index of n that is sandwiched by air and substrate. The interfacial phase shifts of the light wave reflected at the two interfaces are φ_1 and φ_2, respectively. (b) The resonance wavelength variation against the FP thickness for different total interfacial phase shifts of π, $15\pi/9$, $16\pi/9$ evaluated by using Eq. s1. For simplicity, the refractive index of the medium is set as 1.5, and resonance order is m=1. (c) The sensitivity of the first order (m=1), second order (m=2) and third order (m=3) resonance wavelength increases exponentially when the total interfacial phase shift in a round trip approaches 2π. The thickness of the FP cavity is set as 20 nm with refractive index of 1.5.

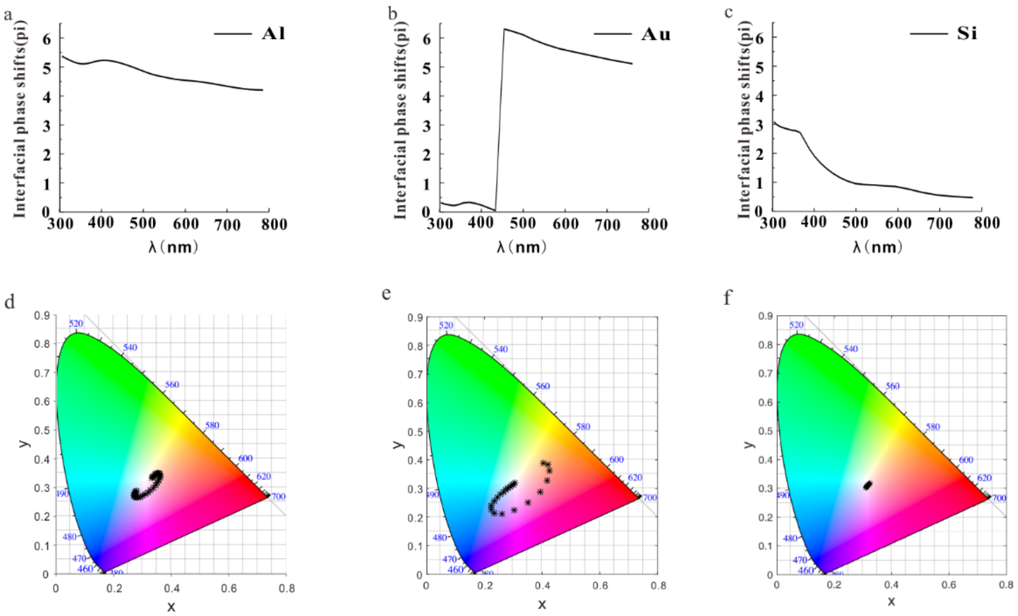

Supplementary Figure 5. The influence of different substrates on the total. The wavelength dependent total interfacial phase shift for MoS_2 on Al (a), Au (b) and Si (c) substrates. It can be seen that the total interfacial phase shifts of the MoS_2-Al configuration are slowly decreasing from 5.5 to 4.17 from 400 nm to 800 nm. The total interfacial phase shifts for the MoS_2-Au configuration drops from near 2π to 5 as the wavelength changes from 400 nm to 800 nm. While integrating MoS_2 layers on the Si substrate, it can be found that the interfacial phase shifts drop rapidly from 3 to around 0 as the wavelength increases from 400 nm to 800 nm. Owing to the different contribution of interfacial phase shifts to the resonance manipulation, MoS_2 thin films with different numbers of layers exhibit wide color appearances. (d - f) Color coordinate diagrams of MoS_2 layers with different nanometric thicknesses prepared on Al (d), Au (e) and Si (f) substrates.

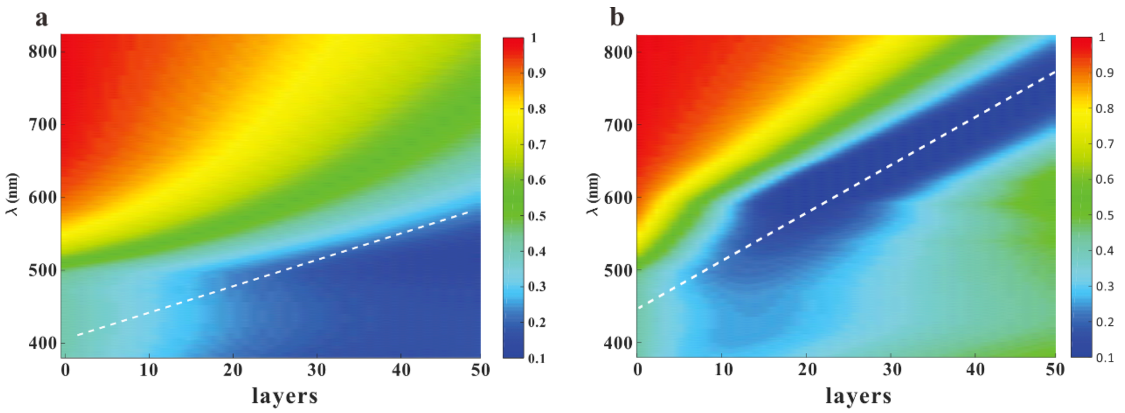

c

Materials	Thickness/monolayer(nm/layer)	Shifted resonance/monolayer (nm/layer)
Ge	0.4	5.95
MoS_2	0.67	13.95
Graphere	0.35	2.32

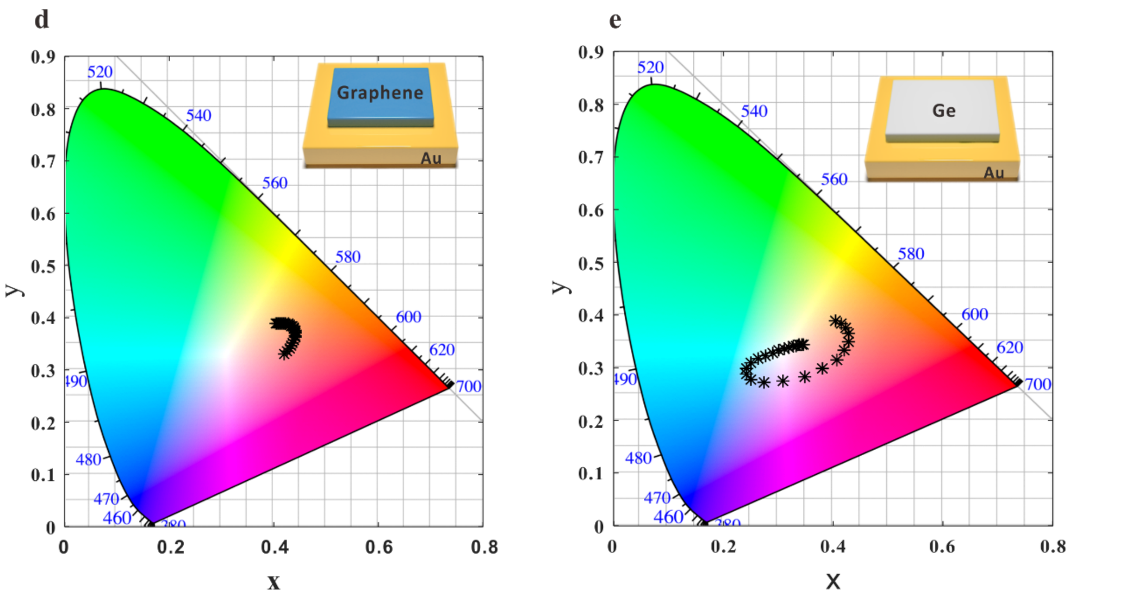

Supplementary Figure 6. The reflectance manipulation of dielectric thin films integrated on the Au substrate by different numbers of layers. The reflectivity from the graphene-Au (a) and Ge-Au structures (b) as a function of the thickness and the incident wavelength. The white dashed lines are guide for eyes illustrating the tunability and sensitivity of resonance wavelengths. A sensitivity of about 2.32 nm per graphene layer and 5.95 nm per Ge[16] layer can be extracted. (c) The comparison among the performance of three kinds of materials on gold substrate. The first column

is the thickness of each layer, and the second and third column are the changing wavelength of each layer thinned and the wavelength of the thinning per nm using MATLAB to calculate. Chromaticity coordinates in the 1931 CIE diagram of the reflected color from graphene (d) and Ge (e) thin film on gold substrate for a variety of thickness.

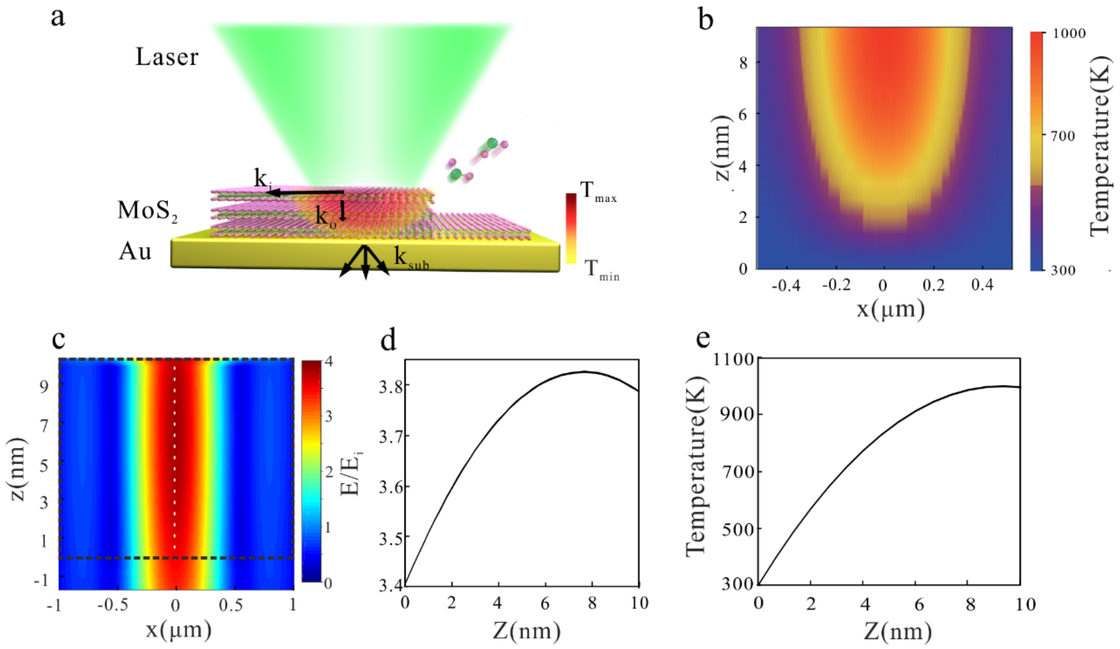

Supplementary Figure 7. Calculated temperature distribution during laser exfoliation. (a) Schematic diagram of laser thinning, k_i and k_o represents in-plane and out-of-plane thermal conductivity, respectively, and k_{sub} represents thermal conductivity between MoS_2 and substrate. (b) The calculated temperature distribution in the XZ plane where the absorbed optical power flow at each point was extracted by the FDTD simulation. (c)The filed distribution within the MoS_2 layers upon focused laser beam illumination. The amplitude is 1.5mV/m. The black dotted line indicates the MoS_2 layer. (d) The electric field intensity at the white dotted line in (c). (e) The vertical direction

plots of temperature distributions, showing a sharp temperature gradient of 74.4 degrees per nm along the stacking layers.

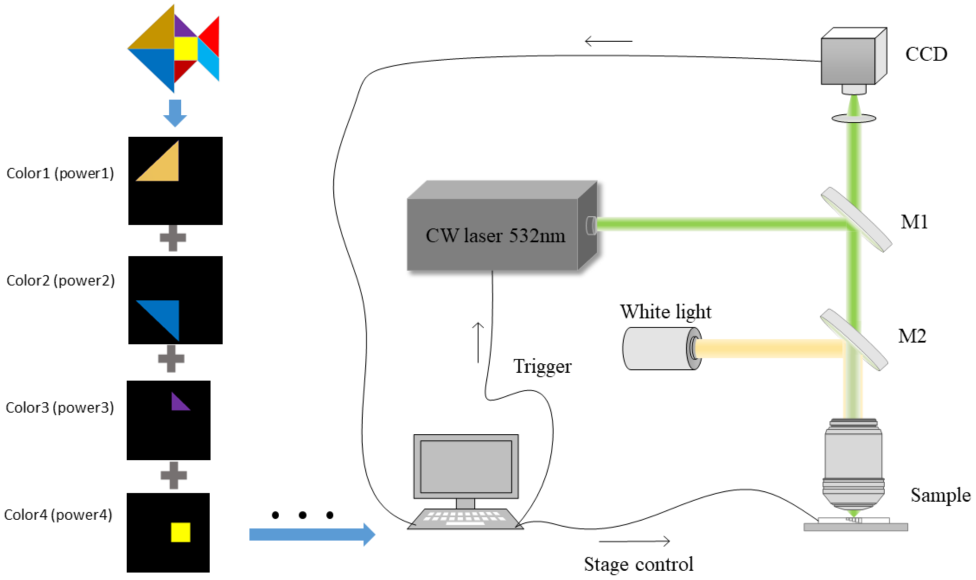

Supplementary Figure 8. The schematic illustration of the experimental system for laser exfoliation and the procedure of laser printing. The designed image is firstly divided into different color regions which are digitalized into gray-level nanometric thicknesses and then printed by different laser powers. The laser beam at the wavelength of 532 nm is focused onto the sample surface by an objective lens with numerical aperture of 0.75 and a lateral pixel size of 0.4 μm. The sample is mounted to a 3D translation stage. The synergistic movement between the focal spot and the 3D stage is controlled by a Labview program (National Instruments Co.).

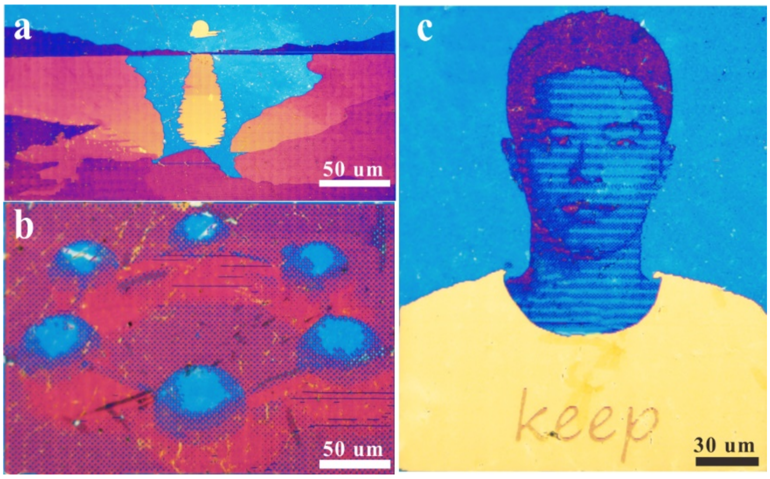

Supplementary Figure 9. Another collection of high definition color prints by the laser exfoliation approach with a high resolution of 58,000 DPI.

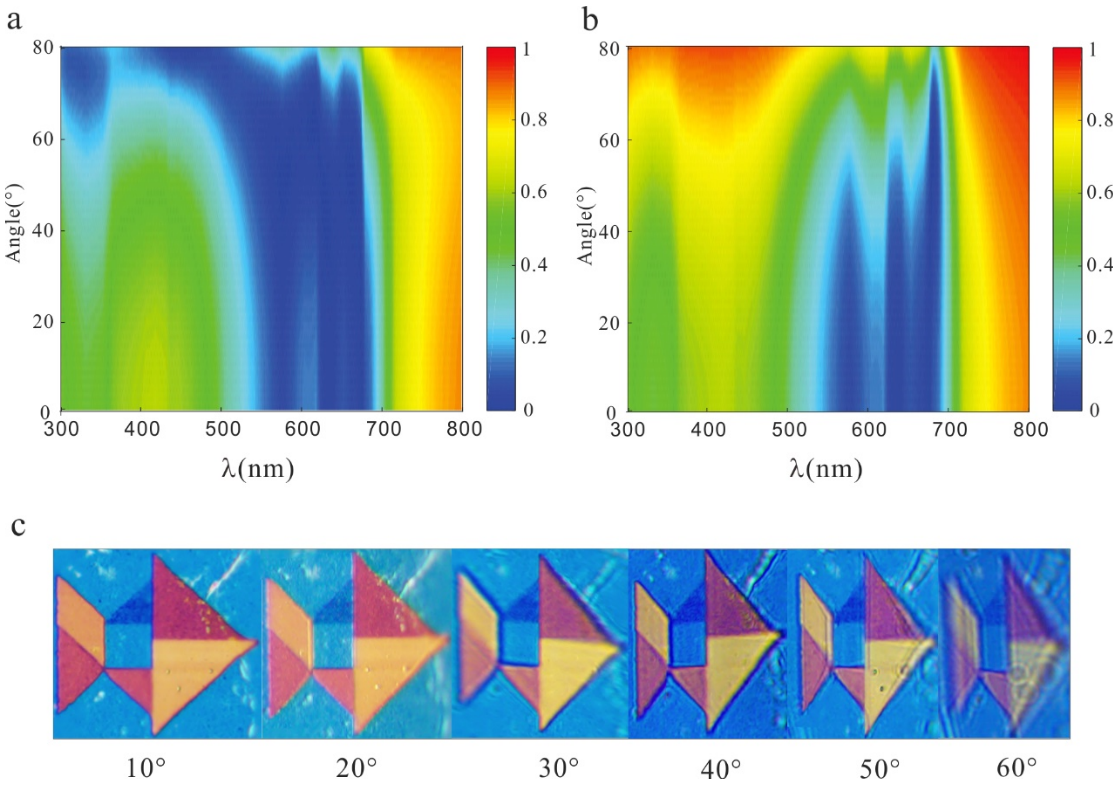

Supplementary Figure 10. Angular dependency of reflectivity spectra. (a,b). The calculated spectra spectra for *s*- and *p*-polarization, respectively, for angles of incidence from 0° to 80° for a 10 nm-thick MoS_2 film integrated on the Au substrate (the value of reflectivity is indicated by the colour bars). (c), Color prints viewed with incident angles from 10° to 60°.

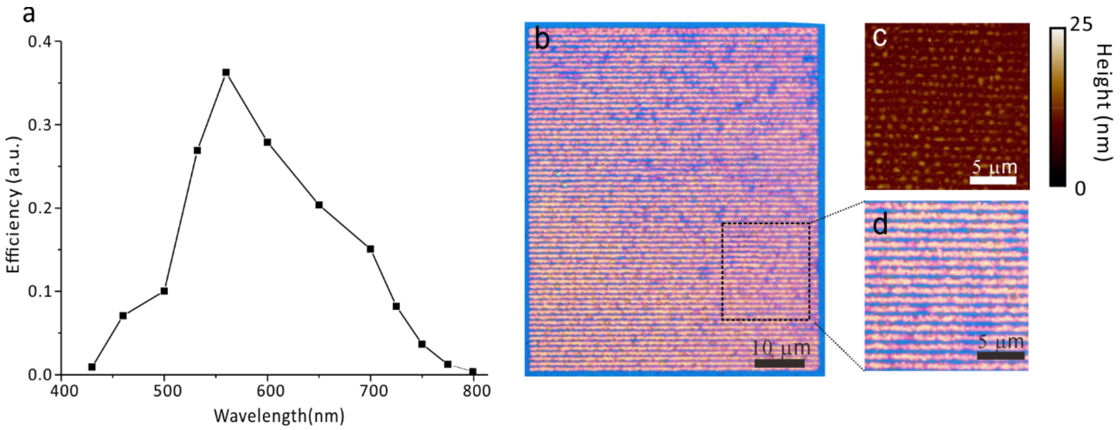

Supplementary Figure 11. Characterization of wavelength dependent diffraction efficiencies of laser exfoliated gratings. (a) Experimentally measured diffraction efficiency (defined as the first-order diffraction intensity divided by the summed intensities of both first-order and zeroth-order diffractions) as a function of wavelengths. (b), Optical microscopic image of the prepared grating with a period of 1000 nm. Atomic force microscope (c) and optical microscopic (d) images of the corresponding gratings that have been stripped directly by the laser beam, respectively.

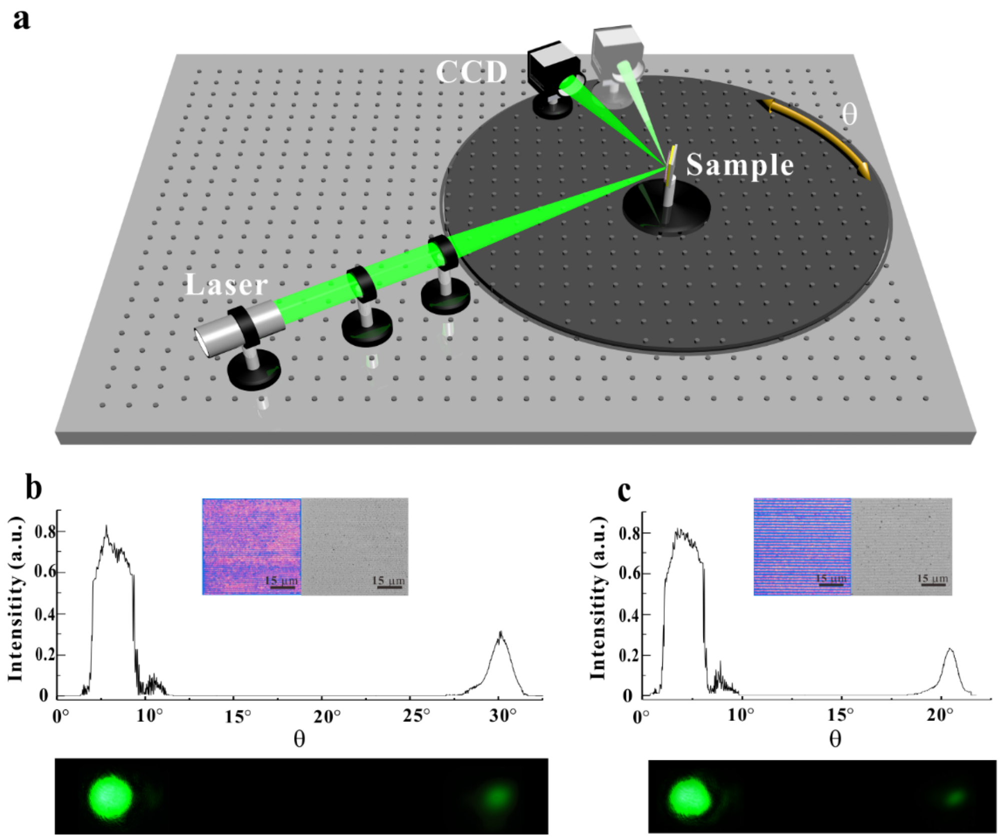

Supplementary Figure 12. Diffraction angle characterization of laser exfoliated nanometric flat gratings with different periods. (a) Experimental configuration for diffraction angle characterization. The grating with a period of 1200 nm (b) and 1800 nm(c) was prepared. At a distance of 5 cm from the sample, CCD was used to acquire the diffraction spot. Diffraction angles were measured to be 26°19' and 17°, respectively, for gratings with 1200 nm and 1800 nm periods. The insets show the optical and SEM images of corresponding gratings exfoliated by direct laser writing.

References

1. Yeh P. *Optical waves in layered media*. Wiley Online Library (1988)
2. Bruna M, Borini S. Optical constants of graphene layers in the visible range. *Applied Physics Letters* **94**, 031901 (2009).
3. Liu H-L, Shen C-C, Su S-H, Hsu C-L, Li M-Y, Li L-J. Optical properties of monolayer transition metal dichalcogenides probed by spectroscopic ellipsometry. *Applied Physics Letters* **105**, 201905 (2014).
4. Ciesielski A, Skowronski L, Pacuski W, Szoplik T. Permittivity of Ge, Te and Se thin films in the 200–1500 nm spectral range. Predicting the segregation effects in silver. *Materials Science in Semiconductor Processing* **81**, 64-67 (2018).
5. Derivaz M*, et al.* Continuous Germanene Layer on Al(111). *Nano letters* **15**, 2510-2516 (2015).
6. Hu L, Shan X, Wu Y, Zhao J, Lu X. Laser Thinning and Patterning of MoS2 with Layer-by-Layer Precision. *Scientific reports* **7**, 15538 (2017).
7. Hu L, Shan X, Wu Y, Zhao J, Lu X. Laser thinning and patterning of MoS 2 with layer-by-layer precision. *Scientific reports* **7**, 1-9 (2017).
8. Gandi AN, Schwingenschlögl U. Thermal conductivity of bulk and monolayer MoS2. *EPL (Europhysics Letters)* **113**, 36002 (2016).
9. Bae JJ*, et al.* Thickness-dependent in-plane thermal conductivity of suspended MoS2 grown by chemical vapor deposition. *Nanoscale* **9**, 2541-2547 (2017).
10. Lugo J, Oliva AI, Riveros H, Ceh O. Heat capacity determination of metallic thin films using temperature profiles at room conditions: Theory. In: *2010 7th International Conference on Electrical Engineering Computing Science and Automatic Control*). IEEE (2010).
11. Touloukian Y, Buyco E. Thermophysical Properties of Matter-The TPRC Data Series. Volume 4. Specific Heat-Metallic Elements and Alloys.). THERMOPHYSICAL AND ELECTRONIC PROPERTIES INFORMATION ANALYSIS CENTER … (1971).
12. Zhu X, Yan W, Levy U, Mortensen NA, Kristensen A. Resonant laser printing of structural colors on high-index dielectric metasurfaces. *Science advances* **3**, e1602487 (2017).
13. Nagasaki Y, Suzuki M, Takahara J. All-Dielectric Dual-Color Pixel with Subwavelength Resolution. *Nano letters* **17**, 7500-7506 (2017).
14. Dong Z*, et al.* Printing Beyond sRGB Color Gamut by Mimicking Silicon Nanostructures in Free-Space. *Nano letters* **17**, 7620-7628 (2017).
15. Aluminum plasmonic metamaterials for structural color printing. *Optics express* **23**, 14552-14560 (2015).
16. Mantina M, Chamberlin AC, Valero R, Cramer CJ, Truhlar DG. Consistent van der Waals Radii for the Whole Main Group. *The Journal of Physical Chemistry A* **113**, 5806-5812 (2009).
